# What Ranges of Probe Pressure Are Applied During Ultrasound Examinations? A Systematic Review

**DOI:** 10.3390/s25113415

**Published:** 2025-05-29

**Authors:** Sławomir Suchoń, Michał Burkacki, Miłosz Chrzan, Mateusz Winder

**Affiliations:** 1 Department of Biomechatronics, Faculty of Biomedical Engineering, Silesian University of Technology, 41-800 Zabrze, Poland; michal.burkacki@polsl.pl (M.B.); milosz.chrzan@polsl.pl (M.C.); 2 Department of Radiodiagnostics, Interventional Radiology and Nuclear Medicine, Faculty of Medical Sciences in Katowice, Medical University of Silesia, 40-055 Katowice, Poland; mwinder@sum.edu.pl

**Keywords:** ultrasound, probe pressure, pressure force, robotic ultrasound systems, diagnostic imaging

## Abstract

The number of US exams has nearly doubled in the last ten years. Many researchers point out the probe pressure force influence on image quality and other aspects of examination. This review aims to identify the range of applied probe pressure during US examinations and gather information on probe compression force values during various US examinations (examination types, body regions, etc.). Methods: A systematic review following PRISMA guidelines was conducted using IEEE Xplore, Web of Science, Scopus, and PubMed/MEDLINE. Studies with quantitative data on probe pressure during US by human operators or RUSs (robotic ultrasound systems) were included. Results: From the 26 included studies, force ranges varied up to 34.5 N for abdominal exams. Robotic systems applied slightly higher maximum forces (34.5 N) than human operators (30 N). Most studies reported positive impacts of force monitoring on image quality and diagnostic precision, with no adverse effects on patient comfort. Conclusions: The evidence collectively emphasizes the critical role of applied pressure in US. The nonuniformity of the reviewed studies does not allow for identifying a clearly defined range of probe pressure forces or force monitoring protocols. Integrating RUS and standardized pressure protocols could improve diagnostic consistency and accuracy.

## 1. Introduction

The number of US exams has nearly doubled in the last ten years [1]. The burden for physicians doing US is increasing as a result of this development. Additionally, this results in a higher number of potential errors connected to body fatigue and a higher incidence of musculoskeletal problems and pain associated with keeping a forced body position [2]. Joint pain is reported by 65.6% of sonographers, and nearly 70% of them associate the occurrence of pain with performing US (another 22% associate it with ultrasound examinations to a moderate degree) [2]. Progress related to automation and the use of artificial intelligence (AI) in industry, science, and many areas of everyday life has been observed for decades. In medicine, however, these are relatively new areas of interest, and the implementation of new technologies is gradual. The use of AI algorithms in education application such as simulators, in simplifying patient access to medical treatment, or in imaging diagnostics is the primary focus of medical sciences [3,4]. Nevertheless, robotics is already in use in a few medical fields where the benefits and capabilities are well established. Such fields include surgery (da Vinci^®^) or interventional cardiology, where robots are used for electrophysiological studies, angioplasty, and ablation procedures (CorPath^®^ 200 Corindus, Stereotaxis Genesis) [5,6]. In the case of US diagnostics, eliminating the human factor at the stage of the mechanical acquisition of ultrasound images through the use of collaborative robots (cobots) could enable more US examinations to be performed, including in a remote and isolated places, e.g., on the battlefield [7,8]. However, acquiring high-quality images is a must for fulfilling those presumptions. It can be rather simple to visualize organs that are placed superficially, like the thyroid, breasts, or lymph nodes. The automated breast ultrasound system (ABUS), a supplemental technique for screening for breast disease, has proven this [9]. Abdominal organs pose a greater challenge due to differences in patient anatomy, including subcutaneous tissue thickness, intraperitoneal fat content, and variability in organ orientation or even location. These differences, both in robotic-assisted and handheld US examinations, require an adjustment of the transducer pressure on the tissue and a variable range of motion in order to obtain high-quality images free of artifacts.

The aim of our work is to analyze the transducer pressure on the tissues during US examinations of superficial and abdominal organs performed by a physician and the robot. The results of the study will be used to determine the range of transducer pressure during US examinations performed autonomously by cobots in future work.

Objective of this systematic review is to answer the research question: What ranges of ultrasound probe pressure are applied during US examinations? Additionally, we aim to address the following questions:

- How does the applied probe pressure differ depending on the type of ultrasound examination (e.g., abdominal and musculoskeletal)?
- Are there significant differences in applied pressure between operators and robotic ultrasound systems (RUSs)?
- Does monitoring the probe pressure during diagnostic examinations affect image quality?
- In which cases is pressure monitoring critical?
- What are the methods for measuring applied pressure?
- What types of force sensors are used?

## 2. Materials and Methods

The PRISMA guidelines [10] were followed in conducting this systematic review. The protocol, PRISMA checklist, and selection data were registered retrospectively in the Open Science Framework (OSF) and are available at https://osf.io/xg4fb (accessed on 1 April 2025).

### 2.1. Eligibility Criteria

Studies that provided data on the measured force or pressure between the US probe and the examination object during US imaging were included. Additional requirements for admission were US operators, inexperienced staff, and robots (RUS) doing examinations on patients or phantoms of human anatomy or simulating it. All examinations (musculoskeletal, cardiology, and abdominal organs) were included; however, dental-related examinations were not. We have separated the articles into several categories. Every technique for measuring pressure or force was covered. Articles that did not give quantitative data on contact pressure or force were disqualified. In silico studies on non-anatomical phantoms, dental-related, veterinary (studies with clinical veterinary objectives), tissue-properties (ARFI), and non-relevant research questions were additional exclusion criteria. Additionally, papers lacking a DOI were not included.

### 2.2. Information Sources and Search Strategy

For our search, we used IEEE Xplore, Web of Science, Scopus, and Pub-Med/MEDLINE. We chose to include Scopus and IEEE Xplore as more engineering-oriented bases because we anticipated finding articles about RUSs.

We selected the following keywords: compression force, contact pressure, ultrasound, sonography, probe pressure, probe force, applied pressure, applied force, force application, force monitoring, and pressure monitoring

Full-text, English-language articles published prior to December 2024 were taken into account. Following prompt was used (adapted each time to the requirements of the selected database): (Ultrasound OR Ultrasonography OR Sonography) AND (Probe pressure OR Probe force OR Applied pressure OR Applied force OR Compression force OR Contact pressure OR Force application OR Force monitoring OR Pressure monitoring) AND (NOT Elastography).

### 2.3. Selection Process

Selection process was made by 3 reviewers using Microsoft Excel 365. After querying the article databases, CSV files with reports including paper metrics were generated. These reports had been uniformed (in column order) and imported to spreadsheet. Duplicates were eliminated using the DOI number as a reference.

**Figure 1:**
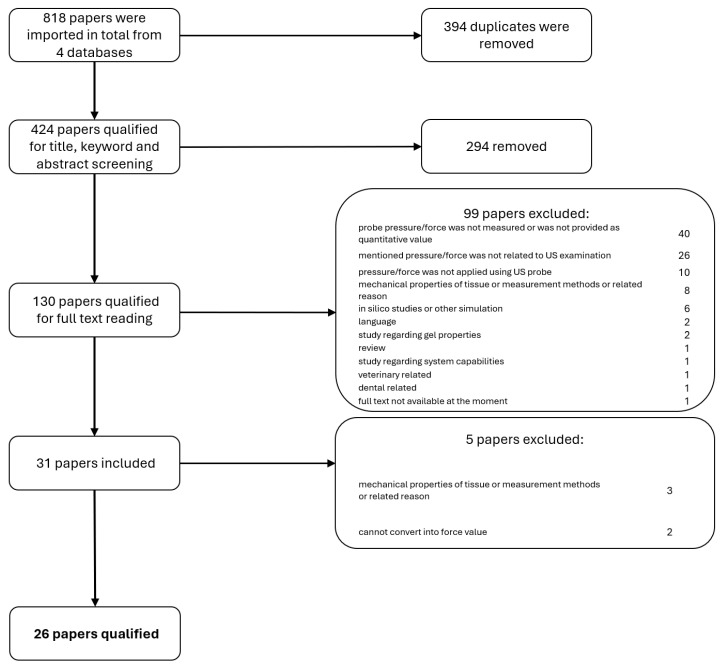
Diagram illustrating the PRISMA workflow, including details on the number of papers removed and excluded at each stage [10].

The following steps are shown in Figure 1. After screening titles, keywords, and abstracts, articles meeting the criteria were selected and further analyzed in full text.

Reviewers were instructed to give comments for each excluded paper. After comments were made by reviewers, the process of standardizing comments was performed using GPT 4o Large Language Model. Please note that the exclusion decision was made only based on reviews, and LLM was used only to improve uniformity of comments and was double-checked.

Zotero (v6) was employed as the reference management system to organize search results, apply tags for inclusion/exclusion decisions, and facilitate citation formatting and bibliographic consistency.

### 2.4. Data Collection and Items

Qualified papers were summarized with a description on following fields: author(s) and year; short study characterization; ultrasound examination type; specific organs; operator (radiologist, sonographer, technician, RUS, etc.); range of applied force or pressure (force/pressure); measurement device; device/sensor placement; positive impact on examination quality; effect on patient comfort and key findings relevant to applied force/pressure. If pressure value was convertible to force, it was performed. Force values were converted to [N] if possible and needed.

### 2.5. Risk of Bias Assessment

Risk of bias in individual studies was not formally assessed. The heterogeneity of study designs, measurement systems, and reported outcomes made the application of standardized bias assessment tools unfeasible. However, major limitations, such as lack of quantitative data or unclear methodology, were noted and considered during synthesis.

### 2.6. Synthesis Methods

Due to methodological heterogeneity among the included studies—particularly in measurement devices, examination types, and reporting units—a narrative synthesis approach was adopted.

Extracted quantitative data were descriptively analyzed. Where possible, pressure values were converted to force units [N]. Force ranges were grouped by examination type and operator (human vs. robotic). Studies reporting diagnostic outcomes were also reviewed to assess relationships between applied force and image quality or patient comfort.

No statistical pooling or meta-analysis was performed.

### 2.7. Reporting

The PRISMA guidelines were followed in conducting this systematic review.

## 3. Results

At the end of the selection process, 26 articles were included, all of which provided quantitative information about the force or pressure of ultrasound (US) probes during examinations performed on patients or anatomical phantoms, either by humans or robotic systems. 

Two articles were excluded because it was not possible to convert the reported pressure values into force values due to insufficient information about the contact area [11,12].

Out of the 26 studies,

- 13 involved examinations conducted by human operators (e.g., sonographers, physicians, or novices).
- 13 involved examinations incorporating robots or other autonomous devices.
- The anatomical focus included 10 studies on abdominal organs, 6 on the cardiovascular system, and 10 on the musculoskeletal system.

Uniformity across studies was poor, likely reflecting the novelty of the topic. The variability reflects differences in the type of ultrasound examinations and methodologies. The dataset included diverse examination types, from musculoskeletal (10 studies) to cardiovascular imaging (6 studies), and abdomen regions like the liver, prostate, and breast (10 studies).

Unfortunately, 40 of the 130 initially screened papers did not provide quantitative values of pressure or force and were excluded [13,14,15,16,17,18,19,20,21,22,23,24,25,26,27,28,29,30,31,32,33,34,35,36,37,38,39,40,41,42,43,44,45,46,47,48,49,50,51,52]. These studies did not have exact measurements but mostly did track force or pressure during ultrasound tests. This omission could suggest that although researchers acknowledged the importance of applied force during tests, they chose to use descriptive distinction (high vs. low force, for instance) because of the measuring difficulties. Other main exclusion reasons were as follows: pressure or force mentioned in paper was not related to US examination; pressure or force measured or applied in experiment was not applied using US probe; paper focused on obtaining mechanical properties of tissue or organ.

None of the studies with human participants proved that probe pressure had any negative effect on patient comfort. This suggests that patients are not burdened by the indicated ranges of applied pressure. Robotic ultrasound systems (RUSs) applied slightly bigger forces, up to 34.5 N [53], than human operators, who applied a maximum force of 30 N [54] (Table 1). During abdominal exams, both groups used the highest pressures, most likely to overcome abdominal cavity resistance or acquire deeper imaging.

Table 2 provides a detailed summary of all included studies, including key methodological details and reported force/pressure values. Table 2 summarizes key data points from the included studies that directly support answering the research questions posed in Section 1. Additionally, it includes other information that the authors deemed relevant for a cross-sectional understanding of study characteristics and methodological diversity. Columns 2 to 5 summarize the methodology used by the researchers, including the study design and population characteristics, the type of ultrasound examination, the target organ, and information about the operator. Column 6 reports the range of applied force or pressure recorded in each study. Columns 7 and 8 describe the measurement devices and sensor placement used by the authors. Columns 9 and 10 address the reported impact on image quality and patient comfort, respectively. Finally, Column 11 summarizes the key findings of each study in relation to applied probe pressure.

## 4. Discussion

### 4.1. Probe Pressure Ranges in Ultrasound Examinations

**Figure 2:**
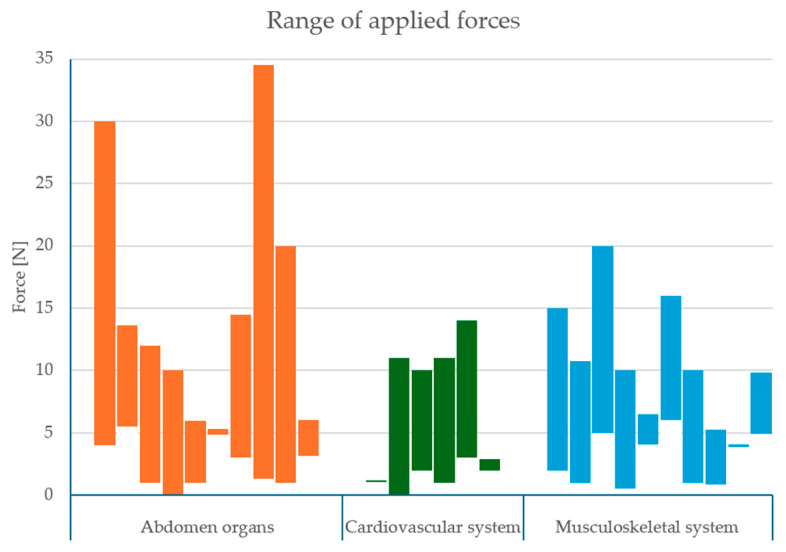
Range of applied forces in each study grouped by body region (as presented in Table 2) [53,54,55,56,57,58,59,60,61,62,63,64,65,66,67,68,69,70,71,72,73,74,75,76,77,78].

The applied pressure of an ultrasound probe varies across selected studies. In the qualified studies, measured probe forces ranged from as low as under 1 N [55,62,63,66,68,70,73,74,75,78] up to 30 N or more [53,54]. For example, in a study of transversus abdominis imaging, the probe force range was 0.88–5.26 N [75], and in a corresponding study, the force was fixed at ~4 N level [76]. Vascular and soft-tissue examinations frequently applied moderate pressures; compression ultrasound for deep veins (e.g., femoral vein) typically required 2–10 N to adequately compress the vessel [67], while measuring carotid artery stiffness involved sweep cycles from ~1 N up to ~11 N [55]. At the other end, certain experiments employed much greater force. Notably, Byenfeldt et al. (2024) found that increasing the probe force from 4 N to about 30 N gives optimal diagnostic performance in liver [54]. In a robotic ultrasound study conducted on prepared canine organs used as in vivo models, the applied contact forces varied depending on the organ, beginning at 1.3 N (for prostate) up to 34.5 N (for targets like liver and pancreas) [53]. These examples illustrate that while most diagnostic exams are conducted with gentle to moderate pressure, the acceptable range can span nearly two orders of magnitude depending on the need. In summary, typical probe pressures are on the order of 2.4–12 N in many settings, but forces three times higher may be encountered in specific applications or experimental setups [53,54]. All of the collected ranges from the included studies grouped by the body region are presented in Figure 2. This wide range underscores the importance of context when comparing applied pressures across ultrasound studies.

### 4.2. Effects of Examination Type on Applied Pressure

The optimal and typical probe pressure is highly dependent on the type of ultrasound examination and the anatomy being imaged. Abdominal imaging often requires moderate pressure for adequate acoustic contact, but the needed force can differ by organ and patient factors. For instance, in liver ultrasound, heavier pressure may improve certain quantitative measures: a study on hepatic steatosis (MASLD) reported that a high probe force (30 N) yielded the best attenuation parameter readings [54]. In contrast, for general abdominal organ imaging (e.g., transabdominal prostate visualization in a telerobotic setup), satisfactory imaging was achieved with only ~3–6 N of force [74]. Pelvic ultrasound offers another example: Schaer (1998) used a controlled perineal probe in urogynecological scanning with forces up to 10 N but emphasized maintaining only gentle contact to avoid patient discomfort [64]. This suggests that abdominal and pelvic exams usually aim for the minimal force that still produces a clear image, with adjustments made for specific techniques.

Vascular ultrasonography presents scenarios where probe pressure is applied deliberately to deform tissues. In compression ultrasound for deep vein thrombosis (DVT) screening, operators applied roughly 2–10 N to compress the femoral or popliteal veins [67]. This range was sufficient to gauge vein compressibility without excessive force that might cause pain. For superficial leg veins, even lower force was needed: one study found that only about 1 N was required on average to collapse a small calf vein in healthy controls (slightly more in varicose vein patients) [62]. Carotid artery scanning for arterial stiffness and blood pressure estimation involved a gradual compression sweep from ~1 N up to 11 N over 10 s [55], indicating a controlled increase to capture tissue response. These examples show that vascular applications commonly use mild to moderate forces; shallow vessels or compliant tissues need only a light touch, whereas deeper or sturdier vessels, especially artery in comparison to vein, need more pressure for diagnostic maneuvers.

Musculoskeletal and superficial tissue imaging generally favors low probe pressures to avoid distorting the anatomy being measured. For example, assessment of abdominal muscle thickness (e.g., transversus abdominis) with ultrasound has been shown to be highly sensitive to probe force. Freehand scanning can inadvertently vary the pressure, so standardized methods enforce a consistent light force (~4 N) to improve measurement reliability [75,76]. In one study, forces under 5 N were sufficient to image the lateral abdominal wall musculature, and applying more pressure only compressed the tissue unnecessarily [75]. In the cervical region, two levels of probe pressure (approximately 4.9 N vs. 9.8 N) were tested for imaging the deep neck flexor muscle. It was found that while both pressure levels yielded acceptable images, maintaining a consistent preset pressure significantly improved the consistency of muscle measurements [77]. Notably, the difference between using ~5 N and ~10 N did not substantially alter the measured dimensions of the muscle (longus colli) as long as the pressure was kept uniform [77]. This indicates that moderate increases within a certain range may not change the imaging outcome, but any variability or excessive force could degrade reproducibility.

### 4.3. Operator Versus Robotic Ultrasound Systems: Pressure Differences

One theme emerging from the review is the difference in pressure application between human operators and robotic ultrasound systems (RUSs). Human sonographers naturally vary in how much force they apply, influenced by experience, technique, and feedback from the patient or image. In contrast, robotic or automated systems can be programmed to apply a consistent predefined force and are equipped with sensors to maintain that target. However, RUS applied force can be tuned with image quality. Several studies highlight that robotic assistance tends to reduce excessive probe force and standardize the contact pressure. For example, Fang et al. compared manual scanning to a co-robotic approach and found that with robotic assistance, the average force dropped from around 20 N (manual) to about 5.5 N [59]. Even with a force constraint, the mean applied force remained lower (13.6 N), improving force stability and image quality [59].

Other studies confirmed that robotic platforms applying controlled forces yielded improved reproducibility and reduced operator dependency, as demonstrated in Virga et al., Duan et al., Zhang et al., and Dall’Alba et al. [56,60,66,70]. Even among human operators, the use of tools like the probe force device (PFD) allowed novices to match the consistency of experienced sonographers, as shown by Kennedy et al. [76]. Jeong et al. also demonstrated reduced inter-rater variability in muscle imaging with calibrated spring gauges [77]. Overall, robotic and assisted systems consistently produced more reliable and repeatable results with lower variability in applied force.

### 4.4. Impact of Probe Pressure Monitoring on Image Quality

Collectively, the evidence strongly indicates that monitoring and controlling probe pressure improves ultrasound image quality and measurement reliability. Pressure-controlled techniques enhanced diagnostic performance across numerous domains: e.g., Ultrasound-Guided Attenuation Parameter measurement in liver [54], elasticity imaging in breast [68], prostate tracking in radiotherapy [61], and deep vein compressibility in DVT screening [67]. In musculoskeletal and vascular imaging, feedback-regulated pressure was associated with lower error and better measurement repeatability [75,76,77].

Excessive or inconsistent force, in contrast, could degrade diagnostic accuracy or produce false results. For instance, fetal MCA Doppler velocities increased with probe pressure, risking an overestimation of fetal anemia risk [65,78]. Several studies noted nonlinear tissue responses at higher force levels (e.g., Sridhar and Insana [73]; Zheng et al. [57]), indicating that image quality benefits are bounded and excessive force may introduce distortions. Monitoring probe pressure mitigates such risks and contributes to standardizing measurement conditions.

### 4.5. Clinical Scenarios Where Pressure Monitoring Is Critical

The review highlights key applications where pressure monitoring is especially critical. These include the following:

- Obstetric imaging, where excessive force may affect fetal hemodynamics or maternal comfort [65,78];
- Elastography and palpation, where force directly influences the diagnostic metrics [57,68,73];
- Vascular compression protocols, where standardized force ensures diagnostic reliability [55,67];
- Robotic/telerobotic ultrasound, where force feedback ensures patient safety and effective automation [56,59,60,66];
- Serial follow-ups and training, where pressure standardization improves reproducibility and quality [75,76,77]; however, lower-experienced operators participating in these studies may be a factor in such improvement.

### 4.6. Methods for Measuring Applied Probe Pressure and Types of Force Sensors Utilized

Force measurement methods in the reviewed studies included a predominantly handheld custom device with multi DOF sensor/load cell (Nano25 ATI, Robotiq FT300) [53,56,58,59,62,66,67,70,71,75,76]; a handheld custom dice with a 1-DOF sensor/load cell [55,57,63,73], or a robotic arm with torque/force sensors (KUKA LBR iiwa R800, UR5) [58,60,61,70,74]. Other methods were measuring pressure with a pressure sensor [65,78]. Other setups like a chair with a pressure-control arm [64], a calibrated spring gauge [77], or other non-conventional set-ups [68,69,72] were incorporated.

Some set-ups were redundant, mostly incorporating robot built-in sensors with an additional sensor in the flange [58,70]. Two systems were supplemented with additional orientation sensors [62,67].

### 4.7. Gaps and Future Research Directions

Despite growing interest, gaps remain. Most notably,

- The lack of standardized force protocols across exam types and patient groups;
- Few large-scale clinical validations of pressure-sensing systems;
- Limited data on patient comfort thresholds and acceptable pressure ranges across populations (none of the 26 studies report patient discomfort);
- Uncertainty about pressure effects in obese patients.

The limitations and gaps discussed above likely stem from the absence of integrated force or pressure sensors in commercial ultrasound (US) systems, apart from research prototypes. In the case of robot-assisted ultrasound (RUS), measurements can be obtained using either integrated sensors in the robot’s joints or additional sensors—typically six-degree-of-freedom (6-DOF) force/torque sensors—mounted on the robot’s flange. Both approaches require a mechanical connection between the robot and the US probe. This connection can be established using a robot gripper, although this solution has certain drawbacks, or by using custom-designed components that ensure a secure and repeatable probe attachment.

In manual ultrasound, the operator holds and manipulates the probe directly, which may result in varying grip patterns. This must be taken into account when designing a handheld measurement system for US probes. If the sensor is placed between the probe and the patient, the operator can still hold the probe directly. However, if the sensor is attached to the probe, direct contact may no longer be possible.

The measuring device should consist of two parts. The first part is an interface for attaching the sensor to the probe, designed to match the shape of the probe and allow the integration of one or more sensors. A challenge here is the organic, non-standardized geometry of ultrasound probes, which varies significantly across types and manufacturers. The second part—connected to the force sensor—should serve as a grip designed for the human hand. One challenge in this part is size and the number of sensors. These factors ultimately define the shape of the handle, which must serve its function as a measuring device while also being ergonomically shaped so as not to compromise the usability and maneuverability of the probe.

An ideal solution would be the use of an ultrasound probe with a factory-integrated force sensor inside of the probe casing, not externally such as the discussed prototypes. Additionally, incorporating a multi-axis force sensor combined with an orientation sensor would be highly beneficial for collecting comprehensive measurement data and monitoring the examination process in terms of applied force range and probe positioning.

Another limitation stemming from non-standardized protocols and varied measurement setups is the inconsistency in sensor placement and orientation. Due to the use of different types of sensors located in various positions, the measured force reported by the authors, if not specified, may reflect either the component normal to the body surface or the component aligned with the axis of the ultrasound probe. Nevertheless, assuming a 10-degree tilt of the probe from a perpendicular orientation to the body surface, the theoretical difference in measured force would be approximately 1.15 N for an applied force of 10 N. This variation appears acceptable as it represents only a small fraction of the reported force ranges (Figure 2).

Future studies should quantify diagnostic performance across varying pressure levels, explore the potential of feedback systems—possibly AI-based—and assess the training benefits of pressure control, particularly for novice users. Standardizing force in clinical ultrasonography could reduce operator dependency, improve inter-study comparability, and enhance diagnostic reliability across care settings.

### 4.8. Trends in Publication Activity

**Figure 3:**
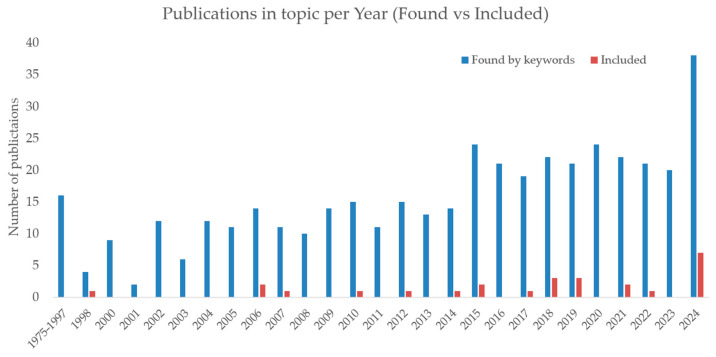
Publications on topic over years: publications found by keyword vs. publications included.

The number of relevant publications over time has been analyzed. As shown in Figure 3, there has been a notable increase in the number of studies addressing probe pressure in ultrasound examinations, particularly in the last decade. In the authors’ opinion, the trend reflects the expanding awareness of the importance of applied pressure for image quality, patient safety, and the standardization of diagnostic protocols. Presented publications on the topic were publications found by keywords (as at stage 2 of PRISMA diagram (Figure 1)) and publications included in studies (final stage of PRISMA diagram).

## 5. Conclusions

The evidence collectively confirms the critical role of applied pressure in ultrasonography, both as a variable that can distort results and as a parameter that can enhance diagnostic precision when standardized. Most of the reviewed studies reported improved image quality or overall examination outcomes, which provides a strong rationale for further exploration of this topic.

However, the methodological heterogeneity of the reviewed literature makes it impossible to define a universal or optimal range of probe pressure. Consequently, no meaningful statistical synthesis can be performed. Despite this, a general range of applied compression forces was identified, reaching up to 34.5 N (in vivo studies with organs). It is important to note that the highest reported force in studies on adult human subjects was 30 N (liver imaging). This value should not be interpreted as a general comfort threshold as tolerance may differ significantly depending on the anatomical region and patient population (e.g., pediatric or geriatric patients). Thus, while the data provide an estimate of expected pressure values and bring us close to “comfort” threshold, they do not allow for defining a universally “safe” threshold.

Standardization of the method should be incorporated in order to compare results among researchers. However, this is hard because there is no designated US system with force measurement, and most researches use their own custom-built prototypes. Implementing engineering solutions such as an US probe with integrated force sensors and eventually orientation tracking can facilitate monitoring of the applied pressure. An ideal design is a probe with factory-built multi-axis force sensors inside its casing, which would be ergonomically optimal and practical for clinical use.

Such solutions enable feedback to the operator, helping maintain a consistent force during examinations and improving reproducibility without disrupting normal workflow. Another important aspect is training benefits with pressure-control tools for novice operators—this could accelerate skill acquisition, and possibly even less-experienced clinicians can perform exams with appropriate and consistent force.

Ultrasound imaging enriched with pressure data could also serve as a valuable source for large-scale data analysis and may be beneficial for AI development in radiology and diagnostic imaging.

In several of the reviewed studies, robotic ultrasound systems (RUSs) played a prominent role. One key advantage is their ability to monitor and limit contact force—crucial for patient safety. Additionally, force sensing offers a method of controlling the scanning process, contributing to the development of fully automated diagnostic workflows. This highlights the importance of integrating force feedback not only as a safety mechanism but also as an enabler of autonomous and intelligent (using quality of acquisition as feedback parameter) ultrasound examinations.

## Figures and Tables

**Table 1:** Highest reported values of pressure, categorized by the type of examination and operator.

	Human Operator	RUS
Abdomen organs	30 N [54]	34.5 N [53]
Cardiovascular system	11 N [55]	14 N [56]
Musculoskeletal system	20 N [57]	16 N [58]

**Table 2:** Summary of included papers with short description.

Reference	Short Study Characterisation	Ultrasound Examination Type	Specific Organs	Operator (Radiologist, Sonographer, Technician, RUS, etc.)	Range of Applied Force or Pressure	(Force/Pressure) Measurement Device	Device/Sensor Placement	Positive Impact on Examination Quality	Effect on Patient Comfort	Key Findings Relevant to Applied Force/Pressure
Byenfeldt et al., 2024 [54]	Prospective diagnostic and experimental study; 60 participants (30 men, 30 women); metabolic-dysfunction-associated steatotic liver disease (MASLD) cohort; control group with no MASLD (BMI < 25)	Quantitative ultrasound-guided attenuation parameter (UGAP)	Liver (convex probe)	Trained operator performing UGAP measurements	Normal force: 4 N; increased force: 30 N	Custom cyclic compression apparatus	On the liver parenchyma; real-time quality map-guided region of interest	Yes	Not specified or not explicitly discussed	Optimal diagnostic performance achieved with 30 N probe force
Fang et al., 2017 [59]	Experimental validation study with human participants and phantom setup; 6 participants (2 expert sonographers, 4 non-experts); experts and non-experts using phantom models for scanning	Robotic-assisted linear ultrasound	Uterus phantom (linear probe)	Expert sonographers and lay participants	Reduced from 20 N to 5.48 N (73%) without constraint; 13.62 N (32%) with force constraint	Handheld US device with dual force sensors: a 6-DOF sensor and a 1-DOF load cell	One sensor on the robotic arm and another on the handheld probe	Yes	Not specified or not explicitly discussed: focused on sonographer (comfort improved)	Co-robotic system reduced human force effort significantly while improving contact force stability and ultrasound image quality
Virga et al., 2018 [60]	Experimental study with robotic platform: 30 volumetric acquisitions on 5 human volunteers; healthy adults aged 26 ± 30 (4 males, 1 female)	Robotic-assisted 3D ultrasound	Soft tissues of the thigh (linear probe)	Robotic system with force sensing; no direct operator involvement	2 N–15 N	Robotic arm (KUKA LBR iiwa R800) with torque sensors and Ultrasonix RP ultrasound machine	Torque sensors integrated into the robotic arm’s joints	Yes	Not specified or not explicitly discussed	Deformation correction improved 3D image quality by compensating for force-induced tissue displacement, enabling more accurate anatomical reconstructions
Schlosser et al., 2012 [61]	Experimental validation study with phantom and in vivo imaging; 5 healthy volunteers and a multimodality pelvic phantom; healthy adults for in vivo imaging; phantom modeling for testing	2D telerobotic ultrasound for prostate monitoring	Prostate (convex probe but not clearly specified)	Robotically assisted imaging; no direct human operator	1 N–12 N	2D Interson 3.5 MHz abdominal ultrasound probe mounted on a robotic manipulator	Infrared tracking for robotic probe localization in six degrees of freedom	Yes	Not specified or not explicitly discussed	Proposed method (with force control) reliably detected prostate displacements
Schimmoeller et al., 2019 [62]	Experimental study on instrumentation design and validation; one subject for in vivo study; phantom and cadaver specimens for testing; in vivo subject: 24-year-old male	Freehand ultrasound imaging	Soft tissue of the arm and leg (linear probe)	Operator using instrumented freehand probe	Minimal force imaging: <1 N; indentation: up to 10.74 N	ACUSON S3000 ultrasound system with Nano25 6-axis load cell and IMU	Probe with integrated load and motion sensors for force and orientation tracking	Yes	Not specified or not explicitly discussed	Instrumented system allowed precise measurement of probe force and orientation, improving reproducibility and enabling minimal force imaging
Mestre et al., 2021 [63]	Deformation correction improved 3D image quality by compensating for force-induced tissue displacement, enabling more accurate anatomical reconstructions	Compression ultrasound using a linear probe	Small saphenous vein (SSV) and deep calf vein (DCV) (linear probe)	Measurements performed by trained clinical researchers	Probe force until vein collapse, median force: 1.03 N (controls)–1.22 N (CVD patients)	Logiq-e system with a 12L-RS linear probe equipped with a force sensor	Probe placed over the small saphenous and deep calf veins at mid-calf height	Not specified	Not specified or not explicitly discussed	Force measurement allows one to determine the hysteresis loop, which reflects the viscoelastic properties of the vessel walls
Zheng et al., 2006 [57]	Experimental in vivo study; 5 male subjects, mean age 29.8 ± 5.1 years; healthy adult males with no neuro-musculoskeletal disorders in their upper limbs	Ultrasound palpation using TUPS system	Transverse carpal ligament (TCL) (hand-held indentation probe)	Trained operators performed the measurements	5 N–20 N	Tissue Ultrasound Palpation System (TUPS) with a 5 MHz transducer and load cell	Probe applied to the palm skin overlying the TCL with positioning based on anatomical landmarks	Not specified	Not specified or not explicitly discussed	Skin-TCL layer was softer than TCL-carpal bone layer: stiffness differences reduced at higher forces
Schaer, 1998 [64]	Experimental clinical study; 30 women (20 with urinary incontinence, 10 healthy controls); women aged 47.6 ± 13.1 years with and without urinary incontinence	Perineal ultrasound with a 5 MHz curved linear array transducer	Bladder base, bladder neck, mid-urethra, and distal urethra (convex probe but not clearly specified)	Clinical researchers using a remote-controlled probe	Probe force adjustable up to 10 N, maintained gentle perineal contact	Custom-designed micturition chair with a pressure-controlled steering arm	Probe positioned against the perineum for sagittal plane imaging	Yes	None of the patients reported discomfort	Bladder neck opening and descent were observable during voiding; 11 women voided without bladder neck descent
Alpert et al., 2015 [65]	Prospective observational study; 29 gravid women (singleton pregnancies); pregnant women in the 2nd to 3rd trimester, median gestational age: 28.5 weeks (range: 22 + 2 to 35 + 6 weeks)	Color and pulsed Doppler ultrasound of fetal middle cerebral artery (MCA) peak systolic velocity (PSV)	Fetal middle cerebral artery (MCA) (convex probe)	Single experienced operator to reduce inter-observer variability	Pressure 1–940 units (~11 N)	GE Voluson E8 ultrasound system with a convex transducer (3.5–5 MHz) equipped with a force sensor	Electronic pressure sensor between the probe and operator’s hand; pressure measured during maternal abdominal scans	Yes	Not specified or not explicitly discussed	Higher applied pressure significantly increased MCA-PSV; excessive pressure could lead to false-positive results in fetal anemia diagnosis
Duan et al., 2024 [66]	Experimental validation of a reinforcement learning approach for robotic ultrasound imaging; tissue phantom experiments for spinal diagnosis using a robotic manipulator; phantom model with embedded spine; no human participants	Ultrasound imaging for spinal diagnosis using a robotic system	Spine (phantom) (linear probe but not clearly specified)	Robotic system autonomously handling ultrasound probe via reinforcement learning	0.5 N–10 N	USB ultrasound probe (Sonoptek) attached to a 6-DoF robotic manipulator with a force/torque sensor (Robotiq FT300)	Probe mounted at the end-effector of the robotic arm, interacting with the phantom’s back	Yes	Phantom-based study	Constraint-aware policy optimization maintained force limits while achieving comparable image quality to unconstrained methods, avoiding excessive force application
Guerrero et al., 2006 [67]	Experimental system evaluation on phantoms and healthy human subjects; phantom experiments and healthy volunteers; phantom models with PVA cryogel vessels mimicking venous structures and healthy human volunteers	Compression ultrasound (CUS) with sensorized probe for deep venous thrombosis (DVT) screening	Deep veins of the lower extremities (e.g., femoral vein, popliteal vein) (linear probe)	Operators using sensorized handheld ultrasound probes	2 N–10 N	Sensorized handheld probe with a 6-DOF force/torque sensor and electromagnetic location sensor	Force/torque sensor integrated into the probe housing; position tracked using an electromagnetic sensor	Not specified	Not specified or not explicitly discussed	The developed method provided an objective measure of venous compressibility, reducing operator dependency and increasing the diagnostic accuracy for DVT screening
Zakrzewski et al., 2018 [55]	Experimental study with real-time algorithm validation; 21 healthy volunteers for single-visit measurements; healthy adults aged 18+	Real-time ultrasound imaging for arterial stiffness and blood pressure estimation	Carotid artery (blood pressure and tissue stiffness assessment) (linear probe)	Operators supervised real-time measurements; volunteers performed self-scans	1 to 11 N over 10 s during compression sweeps	GE Logiq E9 ultrasound system with integrated FUTEK load cell for force measurement	Force sensor mounted on the ultrasound probe’s faceplate, applied to the carotid artery	Yes	Not specified or not explicitly discussed: system aimed at user-guided ease of use	Demonstrated feasibility of non-invasive and calibration-free blood pressure estimation with high reproducibility
Rosen et al., 2024 [68]	Experimental clinical study with force-controlled compression device; 54 female patients with suspicious breast lesions recommended for biopsy; adult women presenting with suspicious breast lesions; 28 lesions malignant, 26 benign	Elasticity imaging with controlled compression during ultrasound imaging	Breast tissue (malignant and benign lesions) (linear probe)	Experienced sonographers performed lesion segmentation and imaging	1 N–6 N	Custom force-instrumented uniaxial compression system integrated with verasonics ultrasound system	Force sensors mounted on a compression plate assembly attached to the ultrasound probe	Yes	Not specified or not explicitly discussed	The study demonstrated that nonlinear elasticity metrics, obtained under controlled forces, can effectively distinguish malignant from benign breast lesions, with significant differences in strain observed
Sahrmann et al., 2024 [69]	Experimental validation of an automated 3D ultrasound system with force control; 8 trials on phantoms with 3 force settings; 10 trials on human tibialis anterior muscle with 2 force settings; custom-designed phantoms; one healthy female subject for tibialis anterior trials	Automated 3D freehand ultrasound (a3DUS) using Aixplorer MACH30 system	Tibialis anterior muscle; muscle-like and cylindrical phantoms (linear probe)	Operators performed freehand trials; automated system for controlled trials	4.1 N–6.5 N	Custom motorized ultrasound probe holder with integrated force control and encoders	Probe mounted on a vertical axis actuated by a direct linear motor; phantom placed on a stable platform	Yes	Not specified or not explicitly discussed	Automated 3D ultrasound system demonstrated higher reproducibility and accuracy than freehand methods, with reduced operator dependency
Zhang et al., 2022 [58]	Experimental validation on phantoms and human trials; phantom experiments: 3 setups; human experiments: 4 healthy volunteers; phantom models and healthy volunteers aged 20–35	Automatic ultrasound scanning system using a robotic mechanism (SAUSS)	Spine (thoracic, lumbar, and thoracolumbar regions) (linear probe)	Robotic system autonomously controlled with minimal operator involvement	Probe contact force: 6–10 N; total resultant force: 14–16 N	Ultrasound probe with integrated force sensor and robotic arm with force control	Probe mounted on a self-adaptive attitude-adjusting mechanism for dorsal scanning	Yes	Visual Analogue Scale (VAS) confirmed no pain reported by volunteers during the scanning process	The SAUSS system maintained stable forces, providing reliable and repeatable images for spinal navigation in minimally invasive surgeries, reducing operator dependency
Zhang et al., 2021 [70]	Experimental validation of a robotic ultrasound system with flexible fixtures; phantom experiments; phantom models of human dorsal anatomy	Robotic ultrasound scanning for minimally invasive spinal surgery navigation	Spine (phantom models for navigation) (linear probe)	Autonomous robotic system with minimal operator involvement	Probe contact force: 1–10 N for safe operation; maximum allowed impact force: 15 N	Robotic arm (UR5) with a flexible fixture and integrated six-axis force/torque sensor	Probe mounted on a flexible fixture equipped with a torsion spring and RGB-D camera	Yes	Phantom-based study	Flexible system improved image consistency and reduced operator dependency; repeatable images showed relative errors of only 4.49% across scans
Tan et al., 2022 [71]	Development and experimental validation of an autonomous ultrasound scanning system; phantom models and validation on bilateral breast regions; no specific human sample size provided; tissue-mimicking phantoms; human anatomical models for breast region testing	Robotic autonomous ultrasound scanning for breast imaging	Breast tissue (phantoms and anatomical models)	System operates autonomously; limited operator involvement	Contact force controlled dynamically within 4.84 N to 5.34 N	RGB-D camera integrated robotic arm equipped with a six-axis force/torque sensor	Ultrasound probe attached to a robotic arm with real-time force and position control	Yes	Phantom-based study	Fully autonomous system reduced scanning variability, enhanced image quality and repeatability, and provided global trajectory coverage within 4 s
Ning et al., 2024 [72]	Development and experimental validation of a lightweight cable-driven robotic ultrasound system; phantom-based testing; no human or live animal samples were involved; tissue-mimicking phantoms; designed for application on human anatomy in future trials, though author presented some results on humans	Cable-driven robotic ultrasound system for multi-region imaging	Designed for imaging multiple organs, including spine, kidney, and breast (linear probe)	System intended for autonomous operation; limited operator intervention during experiments	Force application range: 3–14.5 N, calibrated for safety. The max. comp. force measured for the probe: 14.5 N	Ultrasound probe with cable-driven actuators integrated into the system for precise control	Probe connected to the robotic actuator via a cable-sheath mechanism; body-mounted during testing	Yes	Phantom-based study	Lightweight and portable system demonstrated feasibility for multi-organ imaging with high stability, safety, and adaptability in dynamic environments
Dall’Alba et al., 2024 [56]	Experimental validation of Kernelized Movement Primitives (KMP) for robotic ultrasound; demonstrations using synthetic vascular phantoms and healthy volunteers; synthetic vascular phantoms; healthy volunteers for initial validation	Robotic ultrasound imaging system with interaction force control	Deep veins in the limbs (phantoms) for DVT simulation (linear probe)	Human demonstrators (expert sonographers) provided training data for RUS	Variable force profiles designed for DVT scanning; forces ranged from gentle coupling to compression 3–14 N	Custom recording device integrating a Clarius HD3 Linear probe with force/torque sensors	Force sensor integrated into a 3D-printed adapter mounted on the ultrasound probe	Yes	Not explicitly discussed; focus was on operator performance	KMP-based RUS successfully replicated expert compression patterns, enabling automated DVT assessment
Lediju Bell et al., 2014 [53]	Experimental in vivo study using robotic assistance for ultrasound probe placement; canine model: prostate, liver, pancreas implanted with 3 markers each; canine organs as in vivo models for prostate, liver, and pancreas studies	B-mode ultrasound integrated with robotic positioning for radiation therapy guidance	Prostate, liver, and pancreas (linear probe)	Robotic system autonomously managed probe positioning and tissue deformation reproducibility	Forces ranged from 1.3 N (prostate) to 34.5 N (liver and pancreas) depending on organ and setup	Custom robotic arm equipped with a six-axis force/torque sensor	Probe mounted with a geometric reference for position reproducibility; soft tissue deformations measured	Yes	Not directly applicable; animal model used for evaluation	Position control was more accurate than force control for tissue deformation reproducibility; errors were within acceptable treatment margins for radiation therapy applications
Sridhar and Insana, 2007 [73]	Experimental in vivo study using ultrasonic elasticity imaging; 3 female volunteers (ages 23–28) and 1 patient (age 53) with a fibroadenoma; healthy female volunteers with no history of breast disease and 1 patient with biopsy-confirmed fibroadenoma	B-mode ultrasound using Siemens Sonoline Antares system with VF10-5 linear array transducer	Breast tissue (glandular and fatty regions) (linear probe)	Sonographers manually applied compression with force feedback	1–20 N applied with a strain rate; linear response observed between 2 and 5 N	Ultrasound system equipped with ATI Industrial Automation force sensor for real-time feedback	Force sensor mounted on transducer with a widened contact surface (4 × 8 cmÂ^2^).	Not specified	No discomfort reported	Linear viscoelasticity observed between 2 and 5 N; glandular tissues showed higher viscoelastic response compared to fatty tissues; higher applied forces (>5 N) induced nonlinear and rheodictic behavior
Schlosser et al., 2010 [74]	Experimental study on a telerobotic ultrasound system integrated with a radiotherapy setup; three in vivo volunteer imaging sessions; phantom experiments for tracking; healthy volunteers; phantom models used for testing beam interaction	2D transabdominal ultrasound integrated with radiotherapy linear accelerator	Prostate (primary target for imaging) (convex probe)	Robotic system autonomously controlled with remote haptic operator adjustments as needed	3.15 N–6.01 N	Interson 3.5 MHz abdominal transducer; robotic manipulator with force sensor and haptic control	Transducer mounted on a robotic manipulator designed to minimize collisions with the radiotherapy gantry	Yes	No discomfort reported	The robotic system demonstrated high precision and repeatability, offering significant improvements in volumetric imaging of pelvic organs
Flavell et al., 2019 [75]	Blinded pilot intra-observer reliability study using a novel standardized method for ultrasound imaging; 17 patients with chronic low back pain (CLBP); patients with CLBP aged 18+	Real-time ultrasound imaging with 3.1 MHz curved array probe for TrA thickness measurements	Transversus abdominis muscle (convex probe)	Physiotherapist with 5 years of musculoskeletal ultrasound experience conducted imaging	0.88 N–5.26 N	Probe force device (PFD) attached to a 3.1 MHz ultrasound probe; data recorded at 60 Hz via LabVIEW	Probe positioned lateral to the umbilicus, midway between the iliac crest and lower ribs	Yes	No significant discomfort reported	Standardized ultrasound imaging method demonstrated superior intra-observer reliability compared to freehand methods
Kennedy et al., 2019 [76]	Single-group repeated-measures reliability study comparing freehand and probe force device (PFD) methods; 33 healthy participants (9 males, 24 females); adults aged 18–60 years; excluded if they had low back pain (LBP) in the past year, pregnancy, or other contraindications	Real-time ultrasound imaging with 3.1 MHz curved array probe	Transversus abdominis muscle (convex probe)	Novice examiner with 2 h of prior training conducted imaging and measurements	Force range varied during freehand (uncontrolled) and was standardized in PFD (~4 N)	Probe force device (PFD) integrated with LabVIEW for real-time force and angle monitoring	Probe placed lateral to umbilicus, between the iliac crest and lower ribs, following standardized protocols	Yes	No discomfort reported	PFD method yielded better reliability and lower error in novice examiner’s measurements, especially for contraction phases
Jeong et al., 2015 [77]	Experimental study evaluating intra- and inter-rater reliability of ultrasonography for longus colli muscle under varying probe pressures; 13 participants (11 males, 2 females); university students aged 23.1 ± 2.9 years; healthy adults with no history of neck pain, neuromuscular, or musculoskeletal disorders	Real-time ultrasound with 5–12 MHz linear probe in M-mode	Longus colli muscle (cervical region) (linear probe)	Two experienced examiners with 5 and 2 years of orthopedic physical therapy experience	Inward pressures of 0.5 kg (~4.9 N) and 1.0 kg (~9.8 N) applied using a calibrated spring gauge	Ultrasound system integrated with a hands-free probe holder and spring gauge for consistent pressure application	Probe placed perpendicular to the anterior neck for cross-sectional area and parallel for thickness measurements	Yes	No discomfort reported	Consistent inward pressure significantly improved measurement reliability; differences in CSA and MT were minimal between 0.5 kg and 1.0 kg pressures
Măluțan et al., 2019 [78]	Prospective study assessing the impact of ultrasound probe pressure on fetal Doppler indices; 40 pregnant women with singleton pregnancies, gestational ages between 24 + 0 and 41 + 3 weeks; pregnant women with no pathologies affecting fetal Doppler parameters (e.g., preeclampsia, fetal distress)	US system with 2–9 MHz convex abdominal transducer	Fetal middle cerebral artery (MCA) and surrounding abdominal wall (convex probe)	Examinations conducted by a single experienced obstetrics and gynecology specialist	3 levels of pressure (mean values): 106 gf (~1 N), 206 gf (~2 N), 300 gf (~2.9 N)	Convex transducer integrated with electronic pressure sensor	Probe positioned on the maternal abdomen to assess MCA Doppler indices	No	No discomfort reported; (care taken to avoid prolonged high-pressure applications)	The study highlighted the influence of abdominal pressure on fetal Doppler indices, recommending the use of pressure sensors to standardize measurements and improve diagnostic accuracy

## Data Availability

All data used in this study are available upon reasonable request. Please contact the authors to obtain access. Additionally, all materials related to this systematic review, including the protocol, checklist, and extracted data, are available at the Open Science Framework: https://osf.io/xg4fb (accessed on 1 April 2025).

## Abbreviations

The following abbreviations are used in this manuscript:

US	Ultrasound
RUS	Robotic ultrasound system
